# Acceptance, Usage, and Barriers of Electronic Patient-Reported Outcomes Among German Rheumatologists: Survey Study

**DOI:** 10.2196/18117

**Published:** 2020-07-20

**Authors:** Martin Krusche, Philipp Klemm, Manuel Grahammer, Johanna Mucke, Diana Vossen, Arnd Kleyer, Philipp Sewerin, Johannes Knitza

**Affiliations:** 1 Department of Rheumatology and Clinical Immunology Charité – Universitätsmedizin Berlin Germany; 2 Working Group Young Rheumatology German Society for Rheumatology Berlin Germany; 3 Department of Rheumatology, Immunology, Osteology and Physical Medicine Justus Liebig University Gießen, Campus Kerckhoff Bad Nauheim Germany; 4 Abaton GmbH Berlin Germany; 5 Department of Rheumatology and Hiller Research Unit Rheumatology Heinrich-Heine-University Düsseldorf Düsseldorf Germany; 6 Rheinisches Rheumazentrum Meerbusch St Elisabeth Hospital Meerbusch Germany; 7 Department of Internal Medicine 3 – Rheumatology and Immunology Friedrich-Alexander University Erlangen-Nürnberg University Hospital Erlangen Erlangen Germany

**Keywords:** electronic patient-reported outcome measures, eHealth, rheumatology, rheumatoid arthritis, patient perspective, mobile phone

## Abstract

**Background:**

The use of patient-reported outcomes (PROs) allows for patient-centered, measurable, and transparent care. Electronic PROs (ePROs) have many benefits and hold great potential to improve current usage of PROs, yet limited evidence exists regarding their acceptance, usage, and barriers among rheumatologists.

**Objective:**

This study aims to evaluate the current level of acceptance, usage, and barriers among German rheumatologists regarding the use of ePROs. The importance of different ePRO features for rheumatologists was investigated. Additionally, the most frequently used PROs for patients with rheumatoid arthritis (RA) were identified.

**Methods:**

Data were collected via an online survey consisting of 18 questions. The survey was completed by members of the Working Group Young Rheumatology of the German Society for Rheumatology (Arbeitsgemeinschaft Junge Rheumatologie der Deutschen Gesellschaft für Rheumatologie [DGRh]) at the 2019 annual DGRh conference. Only members currently working in clinical adult rheumatology were eligible to complete the survey.

**Results:**

A total of 119 rheumatologists completed the survey, of which 107 (89.9%) reported collecting PROs in routine practice and 28 (25.5%) already used ePROs. Additionally, 44% (43/97) were planning to switch to ePROs in the near future. The most commonly cited reason for not switching was the unawareness of suitable software solutions. Respondents were asked to rate the features of ePROs on a scale of 0 to 100 (0=unimportant, 100=important). The most important features were automatic score calculation and display (mean 77.50) and simple data transfer to medical reports (mean 76.90). When asked about PROs in RA, the respondents listed pain, morning stiffness, and patient global assessment as the most frequently used PROs.

**Conclusions:**

The potential of ePROs is widely seen and there is great interest in them. Despite this, only a minority of physicians use ePROs, and the main reason for not implementing them was cited as the unawareness of suitable software solutions. Developers, patients, and rheumatologists should work closely together to help realize the full potential of ePROs and ensure a seamless integration into clinical practice.

## Introduction

### Patient-Reported Outcomes in Rheumatology

In order to effectively monitor treatment outcomes, it is crucial to include the patient’s perspective. As quality of life reported by patients and clinical assessment by physicians can have divergent results [1], unaltered, direct patient-reported outcomes (PROs) have become an integral part of today’s clinical routine [2,3] and play a crucial role in clinical studies [4]. The use of PROs allows patient-centered, measurable, and transparent care [3,5]. Various PROs have been established to reflect the individual’s perceived state of health [6]. There are disease-specific PROs, such as the rheumatoid arthritis disease activity index [7], or more general PROs, such as the Health Assessment Questionnaire Disability Index (HAQ-DI) [8]. Various factors, such as symptom severity, physical status, patient satisfaction, and disease-specific comorbidity affect PROs and can be reported. PROs also make it possible to monitor symptoms (eg, fatigue or depression) that are otherwise difficult to measure but play a decisive role in patients’ quality of life [9].

### Treat-to-Target Approach to Increase Therapy Effectiveness

New and innovative therapies have significantly improved treatment results of patients with rheumatic diseases such as rheumatoid arthritis (RA) [10]; however, the effectiveness of new drugs seems to stagnate [11]. To maximize treatment effectiveness, rheumatologists must optimize other therapy variables. For example, the window of opportunity [12], that is, the time frame in which the treatment is most effective, should be respected [13]. In this context, the treat-to-target approach was developed [14]. The aim of this strategy is to define a treatment target at therapy initiation and to closely monitor treatment response in order to identify insufficient treatment success and modify the therapeutic strategy as needed. This approach represents a challenge for rheumatologists, as resources are limited [15]. In reality, therapies are not assessed frequently enough and, therefore, are all too often not adjusted to the current state of the disease [16,17]. Two important reasons for the current poor disease/treatment management are (1) the poor access to rheumatology specialists and (2) an increasing deficit of follow-up appointments for already-diagnosed patients. This situation is likely to worsen due to the current shortage of rheumatologists in Germany [18], and the trend indicates that it will become more difficult in the future [19].

### Electronic Patient-Reported Outcomes on the Rise

Digitalization promises new ways to improve patient outcomes and shape a more efficient and transparent health care environment. Electronic PROs (ePROs) could help to realize the treat-to-target principle on a far larger scale, as the therapeutic outcomes can be evaluated by the patient more frequently and in any setting.

Currently, 49% of German rheumatologists use medical apps in their clinical routine [20]. Various mobile apps offer ePRO services [21,22]. ePROs have the potential to save valuable time and money, as no manual digitization and calculation are required.

Furthermore, ePROs can be recorded anywhere and anytime. Patients can receive reminders to complete the ePROs, which would help reduce the risk of patients being lost to follow-up or failing to complete questionnaires.

Additionally, data would only be collected during disease flare-ups (ie, when patients need to consult their rheumatologists); however, ePROs could also be collected on a regular basis (eg, biweekly) in order to establish benchmarks.

These data would enable rheumatologists to differentiate more precisely between a disease flare-up and a general insufficient response to treatment, thereby allowing treat-to-target strategies to be achievable on a regular basis.

The greatest advantage to digital outcome reporting is the continuous documentation of the individual’s perceived state of health in a standardized, transparent, and validated way. Furthermore, if patients change physicians, they can easily send their data to their new provider (eg, referral to in-house care).

### Aim of This Study

ePROs hold great potential to improve current treatment of rheumatic diseases; however, limited evidence exists regarding usage in clinical practice or hurdles towards acceptance in daily care among rheumatologists. A better understanding of the individual interests, motivations, fears, and circumstances of the treating physician is necessary to promote the further use of ePROs. The aim of this study, therefore, was to identify the current state of ePRO acceptance, usage, and barriers among German rheumatologists.

## Methods

Data were collected via a survey of members of the Working Group Young Rheumatology (Arbeitsgemeinschaft Junge Rheumatologie [AGJR]) of the German Society of Rheumatology at the 2019 annual German Society of Rheumatology conference in Dresden, Germany. The web-based survey was created using SurveyMonkey (SurveyMonkey Inc). The survey was conducted via iPad (Apple Inc) at the AGJR congress booth. Only clinically active adult rheumatologists were asked to complete the survey. Prior to the conference, a task force consisting of AGJR members designed the questionnaire in a web-based consensus meeting after initial individual research of the current literature. Each individual reported the results of their respective deliberations and presented points assessed in the questionnaire that included proposals for the wording of individual questions. In total, 18 questions were formulated assessing acceptance, usage, and barriers concerning ePROs among German rheumatologists.

## Results

In total, 724 members of the German Society of Rheumatology joined the conference and 119 adult rheumatologists participated in the survey. Of the 119 participants, 68 (57.1%) were male and 51 (42.9%) were female. Of the age makeup, 16 of 119 (13.4%) participants were aged between 20 and 30 years; 41 (34.4%), between 30 and 40 years; 23 (19.3%), between 40 and 50 years; 25 (21.1%), between 50 and 60 years; and 14 (11.8%), older than 60 years.

A total of 43 of the 119 (36.1%) participants were residents and 76 (63.9%) were consultants. Of the 43 residents, 35 (81%) were working in a university hospital, 6 (14%) were working in a private or state hospital, and 2 (5%) were employed in a private medical office. Of the 76 consultants, 26 (34%) worked at a university hospital, 21 (28%) worked in a private or state hospital, and 29 (38%) worked in a medical office. These characteristics can be seen in Table 1.

**Table 1:** Characteristics of the respondents.

Characteristics			Respondents, n (%)
**Sex**			
	Female		51 (42.9)
	Male		68 (57.1)
**Age (years)**			
	20-30		16 (13.4)
	31-40		41 (34.4)
	41-50		23 (19.3)
	51-60		25 (21.1)
	>61		14 (11.8)
**Workplace**			
	**Resident**		43 (36.1)
		University hospital	35 (81.4)
		Private or state hospital	6 (13.9)
		Medical office	2 (4.6)
	**Consultant**		76 (63.9)
		University hospital	26 (34.2)
		Private or state hospital	21 (27.6)
		Medical office	29 (38.2)

Of the total participants, 89.9% (107/119) used any form of PRO daily; however, 10.1% (12/119) of respondents indicated that they did not collect PROs at all. Of the rheumatologists using PROs, 77.3% (92/107) of survey participants reported that they used pen and paper–based PROs. Furthermore, 23.5% (28/119) of respondents collected PROs electronically at each patient appointment and 4.2% (5/119) collected PROs electronically before patient contact.

Among respondents not currently using electronic collection, 44% (43/97) reported that they planned to use ePROs; however, 33% (32/97) were still undecided, and 23% (22/97) were not planning to use ePROs (see Table 2).

The question ^“^Why are ePROs not used?” was answered by 68 physicians: 34% (23/68) stated that they did not know a specific software, 12% (8/68) indicated that the introduction of a software was too complicated, and 12% (8/68) thought the software was too expensive. Furthermore, 18% (12/68) of nonimplementers reported that using ePROs was too time-consuming, 16% (11/68) reported that patients preferred paper-based questionnaires, and 32% (22/68) stated “other reasons” (Table 2). Regarding the question “What do rheumatologists use PROs for in routine care?” 66.4 % (79/119) of respondents indicated clinical decision making; 39.5% (47/119), research; 23.5% (28/119), reimbursement; 21.8% (26/119), internal quality monitoring; and 16.8% (20/119), improving patient satisfaction. In addition, 5.9% (7/119) stated that PROs were used for external quality monitoring, and 8.4% (10/119) stated that they were collected but offered no further value (Table 2).

**Table 2:** Participant responses to 4 questions.

Question and answers		Responses, n (%)	
**Are you planning to use ePROs^a^?**			
	Yes		43 (44.3)
	No		22 (22.7)
	Undecided		32 (33)
**Why not?^b^**			
	No software		23 (33.8)
	Implementation too complicated		8 (11.8)
	Patients prefer paper		11 (16.2)
	I prefer paper		2 (2.9)
	Expansive software		8 (11.8)
	No proven benefit		1 (1.5)
	No need		4 (5.9)
	Requires too much time		12 (17.6)
	Other reasons		22 (32.4)
**What do you usually use PROs^c^ for?^b^**			
	No usage		10 (8.4)
	Clinical decisions		79 (66.4)
	Research		47 (39.5)
	Internal quality management		26 (21.9)
	External quality management		7 (5.9)
	To increase patient satisfaction		20 (16.8)
	Reimbursement reasons		28 (23.5)
	Other reasons		7 (5.9)
**Do you have access to the following information before patient contact?^b^**			
	Total score of the recorded PROs		36 (30.3)
	Prevalues		67 (56.3)
	Histograms		11 (9.2)
	Neither		40 (33.6)

^a^ePROs: electronic patient-reported outcomes.

^b^Multiple answers were possible.

^c^PROs: patient-reported outcomes.

**Figure 1:**
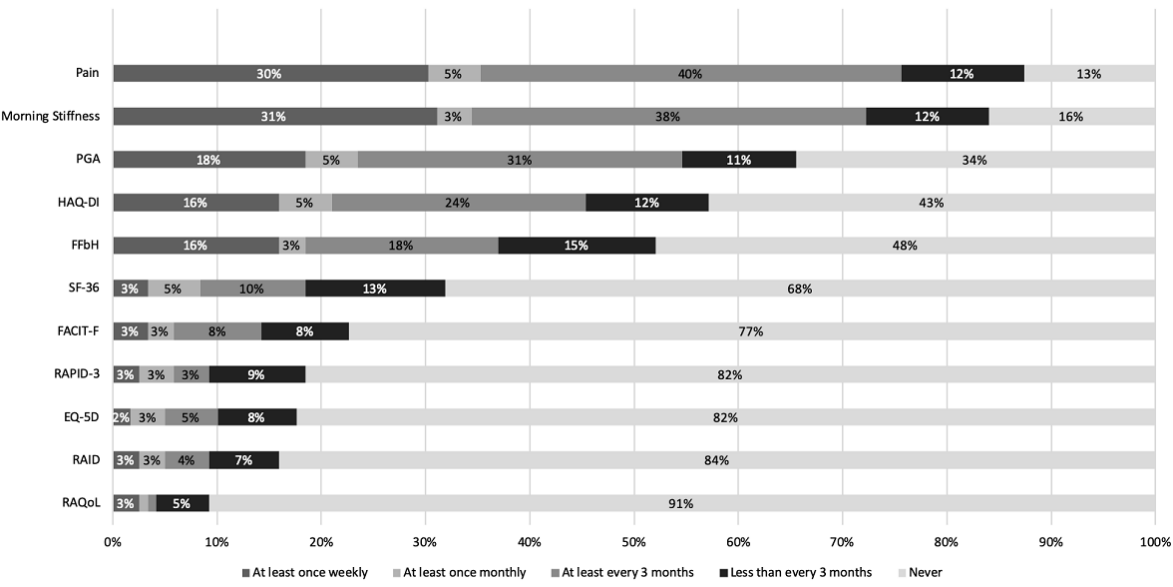
Patient-reported outcomes being used in clinical practice and their respective frequency. EQ-5D: EuroQol Five-Dimensional Questionnaire; FACIT-F: Functional Assessment of Chronic Illness Therapy-Fatigue; FFbH: Funktionsfragebogen Hannover; HAQ-DI: Health Assessment Questionnaire Disability Index; PGA: patient global assessment; RAID: Rheumatoid Arthritis Impact of Disease; RAPID-3: Routine Assessment of Patient Index Data 3; RAQoL: Rheumatoid Arthritis Quality of Life; SF-36: Short Form-36.

The most frequently used PROs for patients with RA were pain, morning stiffness, and patient global assessment, as seen in Figure 1.

In regards to reviewing the results of PROs, 16.8% (20/119) of respondents stated that they never review the results of the PROs before a patient consultation, 29.4% (35/119) stated that they review them sometimes, 31.1% (37/119) stated that they review PROs often, and 22.7% (27/119) claimed to review PROs before every patient visit.

Furthermore, rheumatologists were asked to specify why PROs were not always reviewed. This question was answered by 96 of the 119 (80.6%) participants, and multiple answers were possible. A total of 67% (62/93) of respondents said that this was due to lack of time, 27% (25/93) indicated that PRO results were often not available before the patient appointment, 15% (14/93) answered that the PROs were incompletely answered by the patient, and 12% (11/93) indicated that they trusted their own judgement more than the patient’s. In addition, 12% (11/93) reported that the results of the PROs were often confusing.

We further investigated whether doctors have access to the total score of all recorded PROs, previous PROs, or a histogram of prior values. In total, 30.3% (36/119) of respondents had access to the total score of the recorded PROs, 56.3% (67/119) had access to the previous values of the PROs, 9.2% (11/119) had access to histograms of the PROs, and 33.6% (40/119) did not have access to this information (Table 2). Furthermore, 86.6% (103/119) of rheumatologists reported that their patients did not have access to the results of their PROs. Additionally, we asked how often rheumatologists discussed the results of the PROs with their patients, on a scale of 0 (least often) to 100 (most often), and the mean score was 38.05 (SD 30.57).

Furthermore, the importance of different ePRO functions was analyzed on a scale of 0 to 100. Graphic display of the ePROs was rated with a mean score of 63.50 (SD 31.19). The respondents rated a simple data transfer (eg, from a digital source to the practice’s computer system) a mean score of 76.90 (SD 30.07). Regarding an automatic notification to the physician if a critical threshold value of the ePROs was exceeded, the mean importance was 51.65 (SD 33.5). The notification of the patient if a critical threshold value was exceeded was rated a mean score of 34.55 (SD 30.61), as seen in Table 3.

**Table 3:** Rating of 6 subjects from 0 (lowest agreement) to 100 (highest agreement).

Question	n^a^	Mean (SD)
In how many cases do you discuss the PRO^b^ results with your patients?	119	38.05 (30.57)
How important would the graphic display of ePROs^c^ be to you?	119	63.50 (31.19)
How important would the automatic score calculation and display of ePROs be to you?	119	77.50 (27.64)
How important would the simple transfer of the ePROs to medical reports be to you?	119	76.90 (30.07)
How important would an automatic notification to yourself be for you if a critical threshold is exceeded by an ePRO?	119	51.65 (33.50)
How important would an automatic notification to the patient be for you if a critical threshold is exceeded by an ePRO?	119	34.55 (30.61)

^a^Number of participants who responded to the question.

^b^PRO: patient-reported outcome.

^c^ePROs: electronic patient-reported outcomes.

## Discussion

### Principal Findings

For the first time, this study showed that PROs are widely accepted and used by German rheumatologists; however, it also indicated that the use of ePROs is lagging. Only 10.1% (12/119) of respondents indicated that they do not use PROs at all. The most frequently used PROs were pain (104/119, 87.4%), morning stiffness (100/119, 84.0%), patient global assessment (78/119, 65.5%), HAQ-DI (68/119, 57.1%), and the Hannover functional questionnaire (Funktionalfragebogen Hannover) (62/119, 52.1%). Other validated PRO instruments, such as the Functional Assessment of Chronic Illness Therapy-Fatigue (FACIT-F) were rarely used (27/119, 22.7%). Compared to a previous European survey in 2008 [23], the percentage of rheumatologists in Germany who never use specific PROs was greater in 2019 than in 2008, especially among the use of the HAQ-DI (57% vs 80%) and Short Form-36 (SF-36) (51% vs 32%). These results could have multiple causes. We speculate that our survey consisted of more nonuniversity-based rheumatologists (58/119, 48.7%), who tend to more seldomly use PROs in their daily clinical practice. Interestingly, in 2019, some rheumatologists were using PROs on a weekly basis to allow for more continuous documentation.

The majority (92/119, 77.3%) of German rheumatologists use the traditional pen and paper–based score evaluation, but 23.5% (28/119) already use ePRO in clinical routine. In the future, ePROs will play a more significant role, as 77% (75/97) plan to implement or are undecided about implementing ePROs.

A further barrier stopping German rheumatologists from implementing ePROs is the perception of ePROs being time-consuming processes (12/68, 18%).

The importance of PROs for rheumatologists is reflected by the broad acceptance and use of PROs in clinical care. Only 16.8% (20/119) never review PROs, and the most commonly cited reason (62/93, 67%) for not reviewing the data was a lack of time. More than half (67/119, 56.3%) of the physicians only have access to the last evaluated scores, and only 30.3% (36/119) have access to the complete PRO data set.

A major finding of this study is that discussing the PRO result with the patient was not deemed important. On a rating scale from 0 (lowest importance) to 100 (highest importance) the subject of discussing the result with the patient was rated as a mean of 38.05 (SD 30.57). Additionally, 86.6% (103/119) of the rheumatologists indicated that their patients have no access to their PRO data at all. This indicated a lack of involvement of the patient in therapy and a lack of transparency regarding PROs. Patients’ access to these results and active discussion with their rheumatologist could increase patient satisfaction, adherence, and empowerment, and it could provide a strong basis for better shared decision making (SDM).

The finding that only 9.2% (11/119) of the rheumatologists surveyed have access to PRO graph scores over time, despite the average mean score of 63.50 (SD 31.19) for the importance of having a graphic display of the ePRO scores, highlights the interest in this feature. Interestingly, rheumatologists in Germany prioritize an automatic score calculation with a graphic display over just graphically displaying the results (mean 77.50 vs mean 63.50).

### Comparison With Prior Work

Our data shows a high acceptance of PROs and a lagging use of ePROs. The high usage of traditional PROs in the process of clinical decision making corresponds with the recommendations of the overarching principles of the treat-to-target concept, according to international and European guidelines [24,25]. PROs are being declared as a main treatment target in RA patients [26]. They are particularly important because objective scientific targets make little sense if the patient has subjectively ongoing symptoms. The concept defines quality of life as the primary therapeutic goal; therefore, the primary use of PROs for clinical decision making provides a solid justification for the use of PROs in a treat-to-target approach.

It was recently shown that the vast majority (96%) of patients are willing to share mobile app data for research purposes [27]. Integration of ePROs into existing registries should be actively pursued as a way to generate valuable primary research data and to motivate patients to continue using ePROs [21,28-30].

The main reason for not reviewing and thereby considering the PRO data for treatment is, according to our data, a lack of time (62/93, 67%). The German data align very well with the data from the United States, in which rheumatologists were asked why they do not qualitatively measure RA metrics routinely and 62.5% of respondents stated that a lack of time was the reason [31]. In Israel, 73% of rheumatologists mentioned time constraints as the reason for not implementing the treat-to-target concept [17]. These data are interestingly independent of PRO or clinical metric collection. One end point of future studies should be the time saved by the physician and the patient using ePROs compared with paper and pen–based scores. In addition to the potential time saved by using ePROs, the concept of SDM is an important aspect that could be achieved by continuous patient monitoring and transparent integration of the process into therapy management decisions. A recent study analyzing SDM in RA patients showed that there is still room for improvement [32]. Interestingly, PROs such as pain, morning stiffness, and patient global assessment were much more often compiled than others, such as SF-36, FACIT-F, or computerized adaptive testing. That is certainly due to the simpler data collection (fewer questions) in everyday clinical practice. Less frequently used PROs seem to be collected in clinical trials and have only found their way into clinical practice to a limited degree.

A notable finding of this work is that despite PROs being regularly compiled, a majority of respondents (103/119, 86.6%) stated that their patients have no access to their data. Furthermore, a need to notify patients when values exceed a critical threshold was also given a low score of importance (mean 34.55, SD 30.61). Whether this is also due to a lack of time or whether the physicians trust their own judgement more than the patient's is unclear, but potential approaches for improving patient care can be seen in this study. ePROs could be used as a basis for SDM. It is known that SDM increases adherence and leads to increased patient satisfaction [33]. As the World Health Organization states that 50% of chronically ill patients do not take their medication regularly, adherence and patient satisfaction play a key role in daily rheumatologic practice [34,35].

Particularly among patients, there is great interest in PROs. Navarro-Millán et al [36] showed a marked interest among patients in becoming more involved in the therapy and in collecting and sharing ePROs with their doctors when it facilitated communication. Other publications suggest that patients prefer entering ePROs from home [37]. Walker et al [38] showed high correlation of clinical parameters, like the Simplified Disease Activity Index, Clinical Disease Activity Index, and Disease Activity Score-44, with the Routine Assessment of Patient Index Data 3, and a high willingness of the patients to monitor their targets via a smartphone. The fastest-growing group of smartphone users are older adults [39]. In Germany, 90% of patients with inflammatory rheumatic diseases regularly use a smartphone [27]. Wearable digital technologies enable objective, passive, and continuous ePROs. A growing body of evidence exists that these data can efficiently complement clinical routine procedures [40,41]. ePROs therefore promise to facilitate the realization of the treat-to-target approach.

Various barriers to implementing ePROs have been identified in the survey, among them being the availability and the price of corresponding software. Further difficulties could include the effort of adequate staff and patient training in managing and collecting ePROs.

Regarding the use of PROs for rheumatoid arthritis, our results are in line with previous European results [23].

### Limitations

A total of 119 physicians participated in the survey, which represents approximately 15% of all German rheumatologists [18]. Survey participants tended to be both younger and still in training (rheumatology residents). A high proportion had a university employer, and participants were more often employed in a hospital than the German average. Therefore, the results may be positively biased.

In Germany, PROs are financially reimbursed by the health care system. Consequently, the proportion PROs collected is higher than in other countries [23], and a transfer of the results to other health systems should therefore be carried out cautiously.

Some 32% (22/68) of physicians who reported not using ePROs stated “other reasons” for nonuse. Unfortunately, data security issues were not specifically questioned, though it is also a possible hurdle for the adoption of ePROs. Therefore, the authors suggest that data security should be independently addressed in future research projects.

This question set dealt only with the use of PROs and ePROs, regardless of the method used to determine the outcomes (eg, via tablet in the doctor’s office, or independently via the patient’s smartphone). A distinct connection between the use of PROs and the use/implementation of the treat-to-target concept was not independently questioned; thus, only estimated conclusions can be made about the use of PROs for this concept.

### Potential Vision for the Future

ePROs offer a great potential to overcome current health care obstacles. Automatic score calculations, reminders, and complete data sets save valuable time. Physicians could focus on interpreting the results and getting a preview of the patients, as the results are available before the consultation. In this respect, the treat-to-target approach is not only an option but can also easily be implemented for close monitoring and management (ie, tight control).

An automatic warning system could alert physicians or patients if the PROs exceed or fall below a critical threshold, thereby allowing treatment success to be monitored automatically as a means of supporting the patient and physician. If a patient exceeds the threshold value, a short-term check-up with the physician is recommended and more intensive treatment or a change in therapy may be required. If the patient stays below a threshold value, the next follow-up appointment may not be required as promptly, or the medication may be tapered to allow a more efficient use of limited resources. The recently passed Digital Supply Act in Germany allows physicians to prescribe digital health apps to patients, which are reimbursed by the country’s statutory health insurance. This reimbursement for using apps could foster the wide use of ePROs in clinical routine, as reimbursement appeared to increase usage by almost 10% in a previous study [23].

### Conclusions

Our study results suggest a high acceptance and usage of PROs as a part of patient care in Germany. Nevertheless, some barriers to the usage of PROs were also found to exist, in particular the lack of time for the physician and the absence of data for prior values.

A majority of rheumatologists plan to implement ePROs for clinical decision making. The main barrier to implementing ePROs is a lack of knowledge about suitable software solutions, which matches expectations regarding ease of implementation and price. This work provides a rationale to investigate the effects of ePRO usage in clinical studies analyzing economic and patient outcome as end points.

## Abbreviations

AGJR: Arbeitsgemeinschaft Junge Rheumatologie
ePRO: electronic patient-reported outcome
FACIT-F: Functional Assessment of Chronic Illness Therapy-Fatigue
HAQ-DI: Health Assessment Questionnaire Disability Index
PRO: patient-reported outcome
RA: rheumatoid arthritis
SDM: shared decision making
SF-36: Short Form-36
